# Evaluating the Correlation Between Fecal Microbiota Profiles and Clinical Presentation in Pediatric Patients with Celiac Disease

**DOI:** 10.34172/mejdd.2025.428

**Published:** 2025-07-30

**Authors:** Nastaran Asri, Mohadeseh Mahmoudi Ghehsareh, Fahimeh Sadat Gholam-Mostafaei, Mehdi Azizmohammad Looha, Hamidreza Houri, Somayeh Jahani-Sherafat, Mohammad Rahmanian, Mostafa Rezaei Tavirani, Mohammad Rostami-Nejad

**Affiliations:** ^1^Student Research Committee, Gastroenterology and Liver Diseases Research Center, Research Institute for Gastroenterology and Liver Diseases, Shahid Beheshti University of Medical Sciences, Tehran, Iran; ^2^Celiac Disease and Gluten Related Disorders Research Center, Research Institute for Gastroenterology and Liver Diseases, Shahid Beheshti University of Medical Sciences, Tehran, Iran; ^3^Basic and Molecular Epidemiology of Gastrointestinal Disorders Research Center, Research Institute for Gastroenterology and Liver Diseases, Shahid Beheshti University of Medical Sciences, Tehran, Iran; ^4^Foodborne and Waterborne Diseases Research Center, Research Institute for Gastroenterology and Liver Diseases, Shahid Beheshti University of Medical Sciences, Tehran, Iran; ^5^Laser Application in Medical Sciences Research Center, Shahid Beheshti University of Medical Sciences, Tehran, Iran; ^6^Student Research Committee, School of Medicine, Shahid Beheshti University of Medical Sciences, Tehran, Iran; ^7^Proteomics Research Center, Faculty of Paramedical Sciences, Shahid Beheshti University of Medical Sciences, Tehran, Iran

**Keywords:** Celiac disease, Gut microbiota, Gastrointestinal symptoms, HLA-DQ genotype, Pediatric patients

## Abstract

**Background:**

Celiac disease (CD) is an immune-mediated disorder triggered by gluten in genetically susceptible individuals, often presenting with diverse gastrointestinal and non-gastrointestinal symptoms. Emerging evidence suggests that alterations in gut microbiota composition may influence disease manifestation and clinical outcomes. We investigated the correlation between gut microbiota profiles and clinical features in pediatric patients with CD.

**Methods:**

38 pediatric patients (mean age 10.24 years) with confirmed CD were enrolled from August 2021 to September 2022. We had the data on the relative abundance of various fecal microbiota from our previous study, and collected their clinical and demographic data through questionnaires. Genotyping of human leukocyte antigens (HLA)-DQ2 and HLA-DQ8 alleles was performed.

**Results:**

Significant correlations were found between gut microbiota and both gastrointestinal (GI) and non-GI symptoms. Enterobacteriaceae and *Streptococcus* were positively associated with diarrhea (OR: 5.23 and OR: 3.46), whereas deltaproteobacteria showed a negative correlation (OR: 0.07). Betaproteobacteria and *Staphylococcus* were linked to vomiting (OR: 0.05 and OR: 12.59). Firmicutes and Actinobacteria correlated with weight loss (OR: 2.73 and OR: 16.21), and Verrucomicrobia inversely correlated with fatigue (OR: 0.36). The prevalent HLA genotype DQ2 was correlated positively with Betaproteobacteria (OR: 14.82) and *Prevotella* (OR: 5.05), while *Veillonella* showed a negative correlation with HLA-DQ2 (OR: 0.43).

**Conclusion:**

Overall, the analysis revealed distinct microbiota patterns influenced by symptom presentation and HLA-DQ genotypes, underscoring the need for further research into the therapeutic potential of modifying gut microbiota in managing CD.

## Introduction

 Celiac disease (CD), a common immune-mediated disorder, is caused by the ingestion of gluten in genetically predisposed individuals with human leukocyte antigen haplotype (HLA-DQ2 or HLA-DQ8).^1,2^ It predominantly affects the small intestine by initiating aberrant immune responses, resulting in inflammation, villous atrophy, and damage to the intestinal tissue.^3-6^ CD can manifest with a range of gastrointestinal (GI) symptoms, including abdominal pain, diarrhea, vomiting, bloating, constipation, and weight loss, as well as various non-GI symptoms such as anemia, fatigue, bone disease, neurologic disorders, skin problems, and aphthous stomatitis.^7,8^

 Environmental factors, such as an imbalance in the intestinal gut microbiota profile (dysbiosis), can influence the host’s immune regulation by affecting the growth and homeostasis of the gut’s epithelial layer and mucosal-associated lymphoid tissue.^9,10^ This imbalance plays a pathogenic role in CD, leading to the activation of the patient’s innate immune response and contributing to the development of the disease.^11,12^ An imbalance in the gut microbiota may also occur during the process of CD pathogenesis, leading to the initiation and maintenance of inflammation by allowing harmful bacteria to thrive while reducing the presence of beneficial bacteria.^13^ The establishment of gut microbiota begins in utero or shortly after birth, undergoing significant changes to achieve an adult-like composition by the age of three. This early development is crucial, as dysbiosis during childhood can persist into adulthood, highlighting the importance of acquiring a balanced gut microbiota early on.^14^ Research indicates that pediatric with CD have alterations in their gut microbiota compared to healthy controls.^15^ Specifically, high-risk children for CD exhibit distinct microbiota profiles when compared to those at low genetic risk.^16^ In adults with CD, the most abundant bacterial phylum is Firmicutes, while Proteobacteria are predominantly found in pediatric CD.^16^ The only effective treatment for patients with CD is adherence to a strict gluten-free diet (GFD),^17^ which may induce alterations in the gut microbiota composition due to shifts in the types of dietary constituents consumed.^18,19^

 The genetic makeup of the host plays a crucial role in shaping the composition of the gut microbiome.^20^ The HLA-DQ, as a CD-related genotype, can impact the early composition of gut microbiota. Infants at high genetic risk for CD showed higher levels of Firmicutes and Proteobacteria, and lower levels of Actinobacteria and Bifidobacteria compared to low-risk infants.^3^ Furthermore, high-risk children with the DQ2 gene variant tend to have higher levels of Bacteroidetes and *Enterococcus*, while showing reduced levels of *Clostridium perfringens*, *Parabacteroides*, and *Veillonella*.^21^

 The objective of this study was to explore the potential correlation between the prevalence of bacterial species and the clinical characteristics of pediatric patients with CD.

## Material and Methods

###  Study Design

 Between August 2021 and September 2022, 38 pediatric patients, aged 5 to 15 years (Mean ± SD 10.24 ± 3.46), with confirmed CD (according to the criteria established by the European Society for Pediatric Gastroenterology, Hepatology, and Nutrition (ESPGHAN)^22^ were recruited for this study at the Celiac Disease and Gluten-Related Disorders Research Center of Shahid Beheshti University of Medical Sciences. Among them, the majority (68.42%) adhered to the GFD, of which 42.11% maintained the diet for more than one year. Children who did not cooperate with the researchers or had a history of using specific medications or antibiotics within the last 6 months were excluded from the study. Patients with complicated/refractory CD or those with seronegative CD were also excluded.

###  Data Acquisition

 In our prior investigation, a case-control study was conducted to compare fecal microbiota profiles and intracellular junction gene expression between children with CD and controls. The CD group included patients with active CD and patients on a strict GFD. The control group consisted of individuals with no history of CD or other autoimmune disorders and no first-degree relatives with such conditions. Children who had used specific medications or antibiotics in the 6 months before the study were excluded.^23^ Total genomic DNA was extracted from stool samples, and total RNA was isolated from both whole blood and stool samples, which were subsequently processed for cDNA synthesis. To evaluate the relative abundance of selected bacterial phyla, classes, families, and genera, we employed 16S rRNA-based quantitative PCR (qPCR) with bacterial-specific primers. SYBR Green chemistry was utilized for the qPCR assays, and the relative abundance of bacterial taxa was calculated using a formula that incorporates the efficiency of both universal and taxon-specific primers. We evaluated the relative abundance of fecal microbiota profiles, including Firmicutes, Actinobacteria, Bacteroidetes, Spirochaetota, Verrucomicrobia, and Tenericutes, as well as bacterial classes (Alphaproteobacteria, Betaproteobacteria, Gammaproteobacteria, Deltaproteobacteria), family (Enterobacteriaceae), and genera (i.e.,*Veillonella*, *Ruminococcus*,* Staphylococcus*, *Prevotella*, Clostridiacluster 1,* Fusobacterium*,* Klebsiella*,* Lactobacillus*,* Bifidobacterium*,* Streptococcus*,* Enterococcus*, and* Salmonella*) in these pediatric patients with CD compared with controls. The results showed that the relative abundance of the Firmicutes and Actinobacteria phyla, *Veillonella*, and *Staphylococcus* genera had changed.^24^ The correlation of investigated bacteria with patients’ GI and non-GI symptoms was evaluated in the current study.

 We concurrently collected patients’ clinical and demographic data through a meticulously designed questionnaire administered by a seasoned researcher. This questionnaire encompassed a comprehensive spectrum of inquiries regarding both the demographic and clinical aspects of the patients. Data on clinical aspects, including Marsh classification (Marsh I-III), were obtained from participants in our previous study. The findings revealed that the majority of patients with CD were classified as Marsh III (50%), indicating atrophic changes with villous blunting, while 29% were classified as Marsh II (partial villous atrophy) and 21% as Marsh I (increased intraepithelial lymphocytes without villous atrophy). This demonstrates that a significant proportion of patients with CD exhibited atrophic changes (Marsh II and III). Demographically, we sought information on age, sex, and body mass index (BMI). Clinically, we examined a wide range of symptoms, encompassing GI manifestations such as diarrhea, constipation, vomiting, bloating, abdominal pain, and weight loss, as well as non-GI symptoms including anemia, bone disease, aphthous stomatitis, neurologic disorders, skin afflictions, and fatigue.

###  HLA-DQ2/DQ8 Genotyping

 The HLA DQ2 and DQ8 alleles of patients with CD were assessed using peripheral blood-derived DNA samples by the qPCR technique, following a meticulously established protocol as previously described.^24^

###  Statistical Analysis

 Descriptive statistics were depicted as mean ± standard deviation (SD) for normally distributed numeric variables and as frequency (percentage) for categorical variables. Logistic regression was used to assess the impact of each gut microbiota on the occurrence of various GI or GI symptoms and the presence of HLA DQ2, DQ8, and DQ2/DQ8 alleles. All statistical analyses were conducted using R software (version 4.3.2), with statistical significance set at a threshold of *P* values less than 0.05.

## Results

###  Demographic and Clinical Characteristics of Patients with Celiac Disease

 The study included 38 patients diagnosed with CD, of whom 24 (63.15%) were girls, with a mean age of 10.24 ( ± 3.46) years. Clinical parameters included the presence of other disorders (21.05%) and a previous history of CD (42.11%). Most of the CD patients (68.42%) followed a GFD, of which 42.11% maintained this regimen for over a year.

 Notably, weight loss emerged as the predominant symptom, occurring in 78.95% of cases, followed by fatigue (63.16%) and instances of constipation or abdominal pain (52.63%). The presence of anemia (47.37%) and neurological disorders (42.11%) was also noted, alongside other manifestations such as aphthous stomatitis (42.11%), underscoring the complex clinical spectrum correlated with CD.

###  Correlation of Gut Microbiota with GI and Non-GI Symptoms: Logistic Regression Analysis

 We investigated the correlations between the evaluated gut microbiota and GI and non-GI symptoms of patients. According to the results, Enterobacteriaceae (odds ratio [OR]: 5.23; 95% confidence interval [CI]: 1.55 to 33.49; *P* = 0.027), and *Streptococcus* abundance (OR: 3.46; 95% CI: 1.43 to 10.73; P = 0.013) were positively correlated with an increased risk of diarrhea. Conversely, Deltaproteobacteria abundance was negatively correlated with diarrhea (OR: 0.07; 95% CI: 0 to 0.61; *P* = 0.040). Enterobacteriaceae abundance was negatively correlated with constipation (OR: 0.10; 95% CI: 0.01 to 0.51; *P* = 0.039). Betaproteobacteria abundance was negatively correlated with vomiting (OR: 0.05; 95% CI: 0 to 0.40; *P* = 0.013), while *Staphylococcus* displayed a positive correlation with vomiting (OR: 12.59; 95% CI: 2.09 to 315.56; *P* = 0.050). Clostridia cluster 1 (OR: 0.22; 95% CI: 0.04-0.63; *P* = 0.031) and *Klebsiella* abundance (OR: 0.56; 95% CI: 0.34 to 0.84; *P* = 0.011) were negatively correlated with bloating.

 Regarding non-GI symptoms, Betaproteobacteria abundance was negatively correlated with bone disease (OR: 0.05; 95% CI: 0 to 0.44; *P* = 0.024), while *Staphylococcus* abundance indicated a positive correlation with that (OR: 25.07; 95% CI: 2.56 to 995.67; *P* = 0.031). Firmicutes and Actinobacteria abundance showed a positive correlation with weight loss (OR: 2.73; 95% CI: 1.29 to 8.11; *P* = 0.027 and OR: 16.21; 95% CI: 2.3 to 371.22; *P* = 0.025, respectively). Verrucomicrobia (OR: 0.36; 95% CI: 0.1 to 0.83; *P* = 0.047), Gammaproteobacteria, *Prevotella*, and Deltaproteobacteria abundances were negatively correlated with fatigue, while Tenericutes showed a positive correlation with fatigue (OR: 2.19; 95% CI: 1.15 to 6.06; *P* = 0.044). Lastly, in the domain of skin problems, Firmicutes, Actinobacteria, and *Bifidobacterium* abundances exhibited significant negative correlation (OR: 0.27; 95% CI: 0.07 to 0.65; *P* = 0.017, OR: 0.04; 95% CI: 0 to 0.33; *P* = 0.022, and OR: 0.23; 95% CI: 0.05 to 0.82; *P* = 0.037, respectively), while *Staphylococcus* demonstrated a significant positive correlation (OR: 35.14; 95% CI: 2.96 to 1921.07; *P* = 0.028) (Supplementary file 1, Table S1).

**Figure 1:**
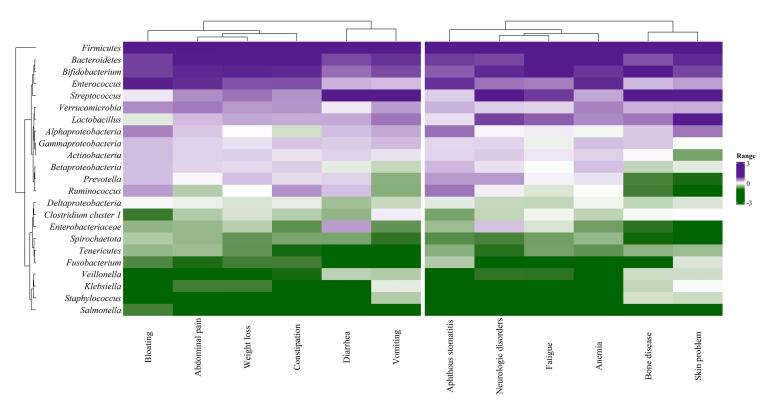
Correlation between fecal microbiota levels and GI and non-GI symptoms in patients with CD

 The examination of microbiota levels in patients with CD showing GI symptoms and those with non-GI symptoms revealed distinct microbiota profiles. The color variations in the heatmap represent bacterial quantities, with purple shades indicating higher levels and green shades indicating lower levels. Notably, Firmicutes consistently showed the highest levels across both groups. Additionally, three other significant microbiota taxa—Bacteroidetes*, Bifidobacterium, *and* Enterococcus*—showed increased levels across this range of symptoms. Conversely, *Salmonella, Staphylococcus, Klebsiella*, and *Veillonella* consistently exhibited lower prevalence across most symptoms (Figure 1).

###  HLA-DQ Genotype Distribution Among Pediatric Patients with CD

**Figure 2:**
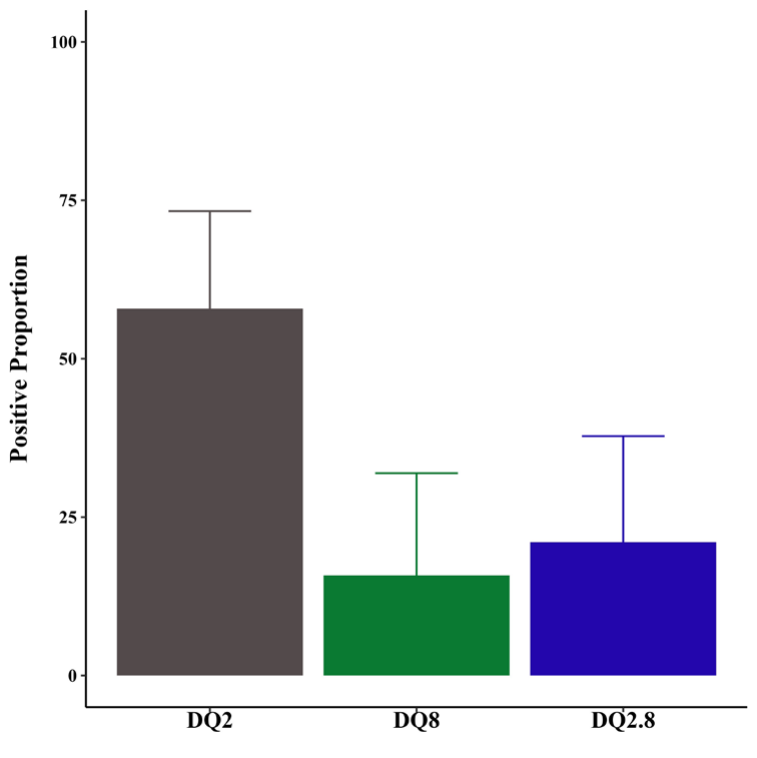
HLA-DQ genotype distribution among pediatric patients with CD

 The distribution of HLA-DQ genotypes among patients with CD is illustrated in Figure 2. The most prevalent genotype was DQ2, detected in 57.89% of cases. In contrast, the DQ8 genotype was present in 15.79% of the patients, while the DQ2.8 genotype was observed in 21.05% of patients with CD.

###  Correlation Between Gut Microbiota and HLA DQ2, DQ8, and DQ2/DQ8 Alleles

 This study investigated the correlations between specific gut microbiota and the presence of HLA DQ2 and DQ8 alleles in patients with CD, as shown in Table 1.

**Table 1:** Correlation Between Gut Microbiota and the Presence of HLA DQ2, DQ8, and DQ2/DQ8 Alleles

**Predictor**	**DQ2**		**DQ8**		**DQ2.8**	
	**OR (95% CI)**	***P*** ** value**	**OR (95% CI)**	***P*** ** value**	**OR (95% CI)**	***P*** ** value**
Enterobacteriaceae	1.06 (0.66,1.67)	0.787	0.75 (0.43,1.39)	0.309	1.15 (0.7,2.27)	0.618
Firmicutes	1.01 (0.65,1.58)	0.972	0.51 (0.18,1.07)	0.125	1.37 (0.83,2.38)	0.228
Actinobacteria	1.32 (0.51,3.67)	0.576	0.26 (0.03,1.29)	0.157	1.33 (0.45,3.97)	0.599
Bacteroidetes	1.2 (0.58,2.64)	0.632	0.67 (0.17,1.96)	0.513	0.99 (0.42,2.17)	0.974
Spirochaetota	0.98 (0.44,2.01)	0.965	0.68 (0.29,1.84)	0.376	1.46 (0.63,5.12)	0.464
Verrucomicrobia	1.03 (0.5,2.26)	0.942	1.46 (0.53,3.59)	0.401	0.7 (0.23,1.61)	0.466
Tenericutes	1.17 (0.66,2.08)	0.584	7.68 (1.34,86.02)	0.047	0.58 (0.29,1.04)	0.077
Betaproteobacteria	14.82 (2.37,167.36)	0.012	0.18 (0.01,1.3)	0.176	0.14 (0.01,0.78)	0.055
*Veillonella*	0.43 (0.14,0.81)	0.046	202.59 (3.72,2828306.78)	0.111	1.45 (0.86,3.13)	0.233
*Ruminococcus*	1.23 (0.83,1.9)	0.304	0.56 (0.32,0.91)	0.024	1.26 (0.79,2.59)	0.410
*Staphylococcus*	0.63 (0.36,0.98)	0.057	1.05 (0.58,2.09)	0.870	1.92 (1.08,4.77)	0.063
Alphaproteobacteria	1.07 (0.76,1.51)	0.668	1.13 (0.7,2.3)	0.667	0.86 (0.6,1.25)	0.422
Gammaproteobacteria	1.25 (0.44,4.43)	0.686	0.58 (0.05,2.72)	0.590	0.98 (0.25,2.97)	0.969
*Prevotella*	5.05 (1.47,33.35)	0.045	N/A	N/A	1.12 (0.63,2.65)	0.728
Clostridia cluster 1	0.49 (0.19,0.95)	0.075	1.27 (0.6,3.32)	0.585	2.09 (1,5.55)	0.090
*Fusobacterium*	0.93 (0.57,1.46)	0.763	2.12 (0.85,9.73)	0.222	0.85 (0.52,1.41)	0.505
*Klebsiella*	0.76 (0.5,1.06)	0.14	2.54 (1.31,10.86)	0.045	0.91 (0.61,1.3)	0.612
*Lactobacillus*	1.07 (0.65,1.76)	0.774	1.06 (0.57,2.62)	0.882	0.9 (0.54,1.57)	0.665
*Bifidobacterium*	2.9 (1.03,9.56)	0.056	1.01 (0.24,5.12)	0.99	0.26 (0.06,0.85)	0.037
*Streptococcus*	0.85 (0.47,1.34)	0.521	2.52 (0.95,8.32)	0.093	0.91 (0.56,1.56)	0.705
*Enterococcus*	4.27 (1.1,30.99)	0.106	0.29 (0.01,1.45)	0.357	0.34 (0.04,1.15)	0.249
Deltaproteobacteria	3.96 (0.91,29.53)	0.113	0.11 (0.00,1.33)	0.147	0.53 (0.08,2.2)	0.433
*Salmonella*	1.32 (0.87,2.06)	0.194	N/A	N/A	1.16 (0.73,1.99)	0.549

Note: Logistic regression was used to assess the impact of each gut microbiota on the presence of HLA DQ2, DQ8, and DQ2/DQ8 alleles. N/A: Not available.

 Betaproteobacteria and *Prevotella* exhibited a positive correlation with HLA-DQ2 alleles (OR: 14.82; 95% CI: 2.37 to 167.36; *P* = 0.012, and OR: 5.05; 95% CI: 1.47 to 33.35; *P* = 0.045, respectively). Conversely, *Veillonella* showed a significant negative correlation with HLA-DQ2 alleles. (OR: 0.43; 95% CI: 0.14 to 0.81; *P* = 0.046).

 Tenericutes and *Klebsiella* demonstrated a positive correlation with HLA-DQ8 alleles (OR: 7.68; 95% CI: 1.34 to 86.02, *P* = 0.047, and OR: 2.54; 95% CI: 1.31 to 10.86; P = 0.045). *Ruminococcus* exhibited a significant negative correlation with HLA-DQ8 alleles (OR: 0.56; 95% CI: 0.32 to 0.91; *P* = 0.024).

 Bifidobacteria emerged as a significant predictor for the presence of HLA DQ2.8 alleles. It displayed a substantial negative correlation (OR: 0.26; 95% CI: 0.06-0.85; *P* = 0.037).

## Discussion

 Gluten can trigger gastrointestinal disorders, and its presence or absence in the diet may alter the diversity and composition of gut microbiota. The GFD is the only effective treatment for diagnosed CD that inevitably impacts the composition and functionality of the gut microbiome. An altered gut microbiome homeostasis, whether as a cause or consequence of the disease, is commonly observed in patients with CD at diagnosis.^25,26^

 According to our findings, an abundance of Enterobacteriaceae was positively correlated with an increased risk of diarrhea and negatively correlated with constipation. The Enterobacteriaceae family comprises both commensal resident bacteria and potentially pathogenic organisms. In a healthy gut microbiota, Enterobacteriaceae typically comprise less than 1% of the total bacterial population. This family encompasses a variety of bacterial genera, including *Escherichia, Enterobacter, *and* Klebsiella*. Among these, *Escherichia coli* has been found to be the predominant species in healthy individuals.^27^ Because *E. coli* strains play a significant role in causing diarrhea,^28^ our correlations suggest it may be possible that increasing levels of Enterobacteria could result in diarrhea and also help resolve constipation. Further research is needed to confirm these findings.

*Klebsiella pneumoniae*, a gram-negative bacterium from the family Enterobacteriaceae, is frequently responsible for antimicrobial-resistant, opportunistic infections in hospital inpatients. *K. pneumoniae* colonization in the human GI tract, especially in patients with inflammatory bowel disease, is well-documented.^29^ Our study showed a negative correlation between *Klebsiella* and bloating, suggesting that higher levels of *Klebsiella* might be correlated with reduced bloating. Gabriela Leite and colleagues examined small intestinal bacterial overgrowth (SIBO) using both culture and high-throughput sequencing methods.^30^ They also employed shotgun sequencing of the luminal small bowel microbiome in a subset of subjects to identify potentially significant bacterial species and strains related to SIBO, including *E. coli* and *Klebsiella*.^30^ Interestingly, a small number of specific strains or species of *E. coli* and *Klebsiella* seem to dominate the microbiome in SIBO and are correlated with varying degrees of abdominal pain, diarrhea, and bloating severity.^31^ Ganji et al studied the imbalance in fecal microbiota and the high prevalence of *Klebsiella* in patients with irritable bowel syndrome (IBS) and gastroenteritis. They discovered that nearly all of the patients with IBS in their study experienced bloating (92.5%).^32^ The overgrowth of certain gram-negative bacteria, including *Klebsiella*, was identified as a potential cause of bloating.^32^ Despite the differences in our findings from past studies, the evidence showing a correlation between bloating and *Klebsiella* bacteria is very important, indicating that investigating this bacteria’s correlation with GI in patients with CD is crucial and requires further research. Furthermore, our results showed that the abundance of *Streptococcus* was positively correlated with diarrhea, suggesting that an increase in this bacterium in patients with CD may lead to more frequent diarrhea.

 Our findings highlight a significant positive relationship between the abundance of Firmicutes and Actinobacteria with weight loss, the most prevalent non-GI symptom in our patients with CD. This indicates that with an increase in the abundance of these bacteria, the level of weight loss may also increase. In this regard, Yu Meng and colleagues conducted a study to explore how different diets impact the microbiota in the small intestine mucus (SIM) and weight regulation in rats. They used correlation analysis to investigate the relationship between weight gain and microbiota in the SIM and feces.^33^ Their study found that the relative abundance of Firmicutes and Actinobacteria in SIM samples did not show a significant correlation with weight gain. Similarly, there was no significant correlation between the relative abundance of Firmicutes and Actinobacteria in fecal samples and weight gain.^33^ Some studies have explored the correlation between the Firmicutes/Bacteroidetes ratio and changes in weight.^32,34,35^ In line with animal research, various human studies have consistently shown that the Firmicutes/Bacteroidetes (F/B) ratio is higher in individuals with obesity and tends to decrease with weight loss.^33-35^ However, other investigations have found significant differences in the F/B ratio between lean and obese individuals.^37-41^ Although our results are not entirely consistent with those of previous studies, they do suggest the important roles of Firmicutes and Actinobacteria in weight loss and gain. Further research using these approaches could contribute to improving weight loss, which is a fundamental issue for patients with CD.

 Furthermore, we found a negative correlation between the abundance of Firmicutes, Bifidobacteria, and Actinobacteria with skin problems. Therefore, the high abundance of Firmicutes, Bifidobacteria, and Actinobacteria, as gram-positive bacteria, may reduce skin problems in patients. Nonetheless, additional research is necessary to fully understand the specific role of microbiota as indicators of skin problems. In all the skin disorders examined in previous studies, a consistent reduction in Bifidobacteria has been observed.^36^ The meta-analysis by Sun and colleagues demonstrated that a combination of *Lactobacillus* and *Bifidobacterium* strains effectively reduces the occurrence of eczema in infants under 3 years old. This suggests that Bifidobacteria could potentially be utilized in patients with CD who experience various skin problems.^37^ The role of Firmicutes remains a source of conflicting and inconsistent findings in many reports. Deng et al discovered that patients with acne have lower levels of Firmicutes compared with the control group.^38^ However, Thompson and others demonstrated that elevated levels of Firmicutes were present in patients with acne before receiving antibiotic treatments.^39^

 Additionally, our results indicate a significant positive correlation between *Staphylococcus* abundance and skin problems. It should be noted that skin *Staphylococcus*, such as *Staphylococcus aureus*, is commonly correlated with various skin disorders.^40^ Currently, there is no evidence to show a correlation between gut *Staphylococcus* and skin problems, especially in patients with CD. Therefore, our results could open up new avenues for investigating these important bacteria.

 Fatigue is one of the non-digestive symptoms that has been frequently reported in our patients. Our results have shown their correlations with different types of bacteria studied. For instance, we found a negative correlation between fatigue and *Prevotella*. Farhadfar et al found that within the gut microbiome, various genera from the Bacteroidetes phylum, such as *Prevotella*, were less abundant in individuals experiencing fatigue.^41^ Fatigue is a significant and frequently reported extraintestinal symptom among patients with CD.^42^ While a GFD may improve fatigue in some cases, its effects remain controversial, as only 50% of adults report improvement after two years on a GFD.^43^ Fatigue is multifactorial and commonly observed in chronic inflammatory and autoimmune diseases, including systemic lupus erythematosus, multiple sclerosis, type 1 diabetes, chronic fatigue syndrome, and rheumatoid arthritis.^44^ In CD, fatigue may be influenced by factors such as anemia, depression, sleep disorders, and thyroid dysfunction, which should be screened for, particularly if symptoms persist despite adherence to a GFD.^43^ While our study identifies potential correlations between fatigue and gut microbiota, these findings must be interpreted cautiously, as fatigue in CD is likely influenced by multiple confounding factors.

 We also conducted genotyping for our patients with CD and explored the correlations between specific gut microbiota and the presence of HLA-DQ2 and DQ8 alleles in such patients. Our findings revealed that a majority of patients were in the DQ2 state, which is the most prevalent allele in patients with CD. Based on our results, there is a negative correlation between the presence of DQ2.8 alleles and the levels of Bifidobacteria. This suggests that patients with both alleles (DQ2.8) are at risk of decreased *Bifidobacterium* levels, which could worsen their CD symptoms and exacerbate the condition. In this regard, the researchers indicated that infants with HLA-DQ2 and HLA-DQ8, as well as first-degree relatives of patients with CD, exhibit higher levels of Firmicutes and Proteobacteria, and lower levels of Actinobacteria and Bifidobacteria. This suggests that the HLA genotype plays a role in the colonization of specific gut bacteria that are more prevalent in patients with CD and their relatives.^41^

 Our analysis revealed more correlations between the composition of bacteria and the presence of various GI and non-GI symptoms in CD. This underscores the critical role of the microbiota in both the amelioration and aggravation of CD symptoms even in cases who are under GFD. If we want to emphasize certain cases for further investigation in subsequent studies based on the results of examining all studied bacteria and their relationships with the common symptoms of CD, Firmicutes are key candidates.

 This study has some limitations that should be acknowledged. First, the sample size of 38 pediatric patients may restrict the generalizability of the findings and limit the statistical power of the analyses. A larger cohort would provide more robust insights and potentially reveal additional correlations. Longitudinal studies are needed to better understand the dynamics of microbiota changes over time and their impact on disease progression and symptomatology. Additionally, while we accounted for various demographic and clinical factors, there may be unmeasured confounders, such as dietary variations beyond the GFD, that could influence microbiota composition and health outcomes.^45^ The reliance on self-reported data from questionnaires also introduces the potential for reporting bias, which may affect the accuracy of symptom prevalence. Finally, the study’s findings may not fully capture the complexity of microbiota interactions and the multifactorial nature of CD, warranting further exploration in future research to validate and extend these observations.

## Conclusion

 We identified distinct variations in the levels of specific bacterial groups in CD patients with GI and non-GI symptoms, suggesting a potential role for bacteria, such as Firmicutes, in shaping the clinical manifestations of the disease. Investigating how a GFD affects microbiota composition and its implications for managing CD deserves more attention. Future studies should aim to elucidate how certain bacterial groups influence the development and presentation of CD, focusing on factors that could be modified for therapeutic purposes. By uncovering the intricate relationships between gut microbiota, symptoms, and genetic markers in CD, we can pave the way for tailored treatment approaches and better outcomes for children with this immune-related condition.

## Supplementary Files

Supplementary file 1 contains Table S1.
