# Supplementary material for: Evaluating the Correlation Between Fecal Microbiota Profiles and Clinical Presentation in Pediatric Patients with Celiac Disease
Source: Middle East J Dig Dis. 2025 Jul 30;17(3):175–82. doi: 10.34172/mejdd.2025.428 (PMC12958314; doi:10.34172/mejdd.2025.428)
Supplement: Supplementary file 1 — contains Table S1. [file mejdd-17-175-s001.pdf]

Middle East J Dig Dis. July 2025;17(3):175-S1

10.34172/mejdd.2025.428
http://mejdd.org

Table S1. Correlation of Various Gut Microbiota with GI and non-GI Symptoms

Predictor	Diarrhea		Constipation		Vomit		Bloating		Abdominal pain		Anemia		Bone disease		Aphthous stomatitis		Weight loss		Fatigue		Neurologic disorders		Skin problems	
	OR (95% CI)	p	OR (95% CI)	p	OR (95% CI)	p	OR (95% CI)	p	OR (95% CI)	p	OR (95% CI)	p	OR (95% CI)	p	OR (95% CI)	p	OR (95% CI)	p	OR (95% CI)	p	OR (95% CI)	p	OR (95% CI)	p
Enterobacteriaceae	5.23 (1.55 ,33.49)	0.027	0.1 (0.01 ,0.51)	0.039	0.79 (0.49 ,1.29)	0.320	0.83 (0.52 ,1.33)	0.411	0.79 (0.45 ,1.23)	0.329	0.56 (0.22 ,0.97)	0.097	0.72 (0.43 ,1.22)	0.196	0.84 (0.52 ,1.31)	0.447	0.87 (0.39 ,1.52)	0.68	1.22 (0.78 ,1.98)	0.373	5.02 (1.51 ,40.79)	0.053	0.73 (0.44 ,1.23)	0.211
Firmicutes	1.14 (0.69 ,1.93)	0.597	1.04 (0.68 ,1.62)	0.849	0.77 (0.44 ,1.27)	0.325	1.51 (0.94 ,2.64)	0.109	1.26 (0.83 ,2.01)	0.293	1.09 (0.71 ,1.7)	0.683	0.85 (0.46 ,1.48)	0.569	1.36 (0.88 ,2.22)	0.179	2.73 (1.29 ,8.11)	0.027	1.03 (0.66 ,1.61)	0.91	1.1 (0.71 ,1.71)	0.678	0.27 (0.07 ,0.65)	0.017
Actinobacteria	1.5 (0.51 ,4.68)	0.462	1.12 (0.44 ,2.94)	0.812	0.91 (0.29 ,2.69)	0.865	2.63 (0.93 ,8.78)	0.084	1.72 (0.68 ,4.8)	0.265	1.61 (0.64 ,4.4)	0.327	0.66 (0.17 ,2.22)	0.515	1.65 (0.65 ,4.54)	0.304	16.21 (2.3 ,371.22)	0.025	1.03 (0.39 ,2.75)	0.959	2.05 (0.79 ,5.93)	0.155	0.04 (0 ,0.33)	0.022
Bacteroidetes	0.71 (0.29 ,1.43)	0.378	2.06 (1.05 ,5.05)	0.063	0.6 (0.23 ,1.26)	0.229	0.87 (0.42 ,1.64)	0.674	0.88 (0.48 ,1.58)	0.674	1.03 (0.57 ,1.88)	0.918	0.45 (0.13 ,1.14)	0.152	0.81 (0.42 ,1.46)	0.497	1.27 (0.59 ,3.26)	0.575	0.77 (0.41 ,1.41)	0.392	0.71 (0.35 ,1.3)	0.298	0.43 (0.11 ,1.09)	0.134
Spirochaetota	1.3 (0.58 ,4.25)	0.593	1.57 (0.77 ,4.04)	0.253	0.82 (0.38 ,1.92)	0.611	3.82 (1.09 ,21.26)	0.083	2.84 (1.09 ,13.85)	0.098	3.09 (1.14 ,16.42)	0.091	0.73 (0.33 ,1.77)	0.426	0.8 (0.36 ,1.62)	0.529	0.93 (0.28 ,2.12)	0.883	1.63 (0.79 ,4.18)	0.213	0.66 (0.26 ,1.34)	0.283	0.71 (0.31 ,1.68)	0.375
Verrucomicrobia	0.54 (0.16 ,1.33)	0.257	1.23 (0.62 ,2.74)	0.565	0.91 (0.36 ,1.93)	0.819	1.44 (0.71 ,3.16)	0.308	2.03 (0.94 ,5.73)	0.117	1.52 (0.76 ,3.64)	0.274	0.74 (0.22 ,1.77)	0.564	0.98 (0.47 ,1.93)	0.949	2.26 (0.77 ,10.08)	0.22	0.36 (0.1 ,0.83)	0.047	0.67 (0.27 ,1.36)	0.312	0.66 (0.18 ,1.65)	0.454
Tenericutes	0.58 (0.29 ,1.05)	0.079	0.72 (0.33 ,1.28)	0.308	0.74 (0.4 ,1.36)	0.307	1.81 (0.87 ,5.78)	0.205	4.91 (1.54 ,38.85)	0.056	1.31 (0.76 ,2.5)	0.353	1.84 (0.76 ,7.58)	0.302	1.82 (0.94 ,5.11)	0.141	1.68 (0.9 ,3.27)	0.098	2.19 (1.15 ,6.06)	0.044	0.78 (0.43 ,1.36)	0.389	3.13 (0.96 ,16.88)	0.136
Betaproteobacteria	0.31 (0.05 ,1.18)	0.159	1.26 (0.55 ,3.51)	0.601	0.05 (0 ,0.4)	0.013	1.59 (0.66 ,4.29)	0.295	1.12 (0.49 ,2.81)	0.783	1.54 (0.66 ,4.6)	0.355	0.05 (0 ,0.44)	0.024	2.25 (0.91 ,8.19)	0.130	1.18 (0.43 ,5.02)	0.782	0.25 (0.04 ,0.78)	0.058	0.98 (0.39 ,2.29)	0.968	0.25 (0.03 ,1.2)	0.154
Veillonella	2.73 (1.15 ,12.31)	0.104	1.07 (0.7 ,1.62)	0.751	2.3 (1.08 ,8.91)	0.115	0.74 (0.47 ,1.14)	0.169	0.81 (0.51 ,1.23)	0.340	0.83 (0.53 ,1.25)	0.390	2.99 (1.08 ,17.64)	0.141	0.64 (0.39 ,0.98)	0.050	0.42 (0.09 ,1)	0.166	1.28 (0.84 ,1.98)	0.247	1.05 (0.7 ,1.63)	0.808	3.69 (1.16 ,24.94)	0.115
Ruminococcus	1.09 (0.71 ,1.95)	0.733	1.4 (0.94 ,2.39)	0.125	0.73 (0.47 ,1.1)	0.126	1.34 (0.84 ,2.74)	0.304	0.42 (0.12 ,0.83)	0.074	0.84 (0.52 ,1.23)	0.395	0.67 (0.42 ,1.03)	0.069	1.93 (1.08 ,5.32)	0.098	0.39 (0.11 ,0.99)	0.117	0.52 (0.18 ,0.95)	0.114	0.96 (0.65 ,1.43)	0.817	0.66 (0.41 ,1.01)	0.059
Staphylococcus	1.31 (0.83 ,2.24)	0.278	1.09 (0.74 ,1.61)	0.654	12.59 (2.09 ,315.36)	0.050	0.97 (0.64 ,1.48)	0.894	0.95 (0.65 ,1.39)	0.791	1.35 (0.92 ,2.05)	0.131	25.07 (2.56 ,995.67)	0.031	0.97 (0.66 ,1.42)	0.861	0.41 (0.1 ,0.85)	0.077	1.16 (0.78 ,1.74)	0.465	1.01 (0.69 ,1.5)	0.94	35.14 (2.96 ,1921.07)	0.028
Alphaproteobacteria	1.22 (0.82 ,2.15)	0.386	0.91 (0.63 ,1.27)	0.589	1.21 (0.81 ,2.11)	0.406	1.72 (1.04 ,3.93)	0.098	1.28 (0.92 ,1.91)	0.169	1.02 (0.73 ,1.43)	0.883	1.12 (0.73 ,1.98)	0.645	2.39 (1.27 ,7.45)	0.049	1.22 (0.81 ,1.81)	0.308	1.17 (0.84 ,1.67)	0.347	1.09 (0.79 ,1.59)	0.603	1.25 (0.79 ,2.43)	0.418
Gammaproteobacteria	1.47 (0.48 ,4.36)	0.465	2.82 (0.9 ,13.32)	0.126	1.39 (0.45 ,4.08)	0.534	2.37 (0.85 ,8.8)	0.125	1.53 (0.59 ,4.88)	0.410	2.59 (0.88 ,11.19)	0.132	1.15 (0.29 ,3.55)	0.812	1.49 (0.57 ,4.43)	0.419	1.22 (0.38 ,5.94)	0.764	0.06 (0 ,0.39)	0.016	1.46 (0.56 ,4.3)	0.441	0.41 (0.06 ,1.7)	0.31
Prevotella	0.92 (0.53 ,1.83)	0.780	0.66 (0.25 ,1.21)	0.279	0.41 (0.09 ,0.84)	0.089	1.04 (0.61 ,2.12)	0.895	0.44 (0.13 ,0.97)	0.129	0.66 (0.26 ,1.18)	0.251	0.38 (0.07 ,0.79)	0.076	1.42 (0.8 ,3.47)	0.324	0.93 (0.35 ,1.71)	0.848	0.17 (0.03 ,0.63)	0.021	1.37 (0.78 ,3.23)	0.357	0.42 (0.1 ,0.83)	0.064
Clostridia Cluster 1	0.77 (0.41 ,1.36)	0.350	0.76 (0.4 ,1.27)	0.329	1.28 (0.71 ,2.69)	0.456	0.22 (0.04 ,0.63)	0.031	0.75 (0.4 ,1.25)	0.307	0.84 (0.47 ,1.39)	0.514	1.12 (0.6 ,2.4)	0.749	0.47 (0.18 ,0.89)	0.058	1 (0.48 ,1.85)	0.992	1.3 (0.77 ,2.37)	0.332	0.89 (0.52 ,1.48)	0.643	1.14 (0.6 ,2.46)	0.715
Fusobacterium	0.82 (0.5 ,1.37)	0.432	1.1 (0.7 ,1.73)	0.664	0.96 (0.59 ,1.67)	0.878	1.04 (0.65 ,1.76)	0.883	0.9 (0.57 ,1.4)	0.649	0.64 (0.35 ,1.03)	0.098	0.79 (0.46 ,1.38)	0.378	2.24 (1.2 ,5.7)	0.038	1.14 (0.63 ,1.94)	0.635	0.79 (0.45 ,1.27)	0.366	0.24 (0.04 ,0.57)	0.021	2.87 (1.1 ,12.02)	0.09
Klebsiella	0.97 (0.67 ,1.36)	0.848	0.87 (0.62 ,1.17)	0.357	1.34 (0.94 ,2.08)	0.130	0.56 (0.34 ,0.84)	0.011	1.05 (0.78 ,1.43)	0.732	0.88 (0.64 ,1.19)	0.425	1.23 (0.84 ,1.89)	0.282	0.55 (0.33 ,0.81)	0.008	1.13 (0.77 ,1.74)	0.563	0.94 (0.68 ,1.28)	0.691	0.93 (0.68 ,1.26)	0.646	1.32 (0.9 ,2.09)	0.168

Supplementary file 1

Lactobacillus	0.91 (0.55 ,1.63)	0.712	0.71 (0.33 ,1.2)	0.281	0.97 (0.59 ,1.81)	0.918	0.46 (0.12 ,0.89)	0.101	0.66 (0.28 ,1.12)	0.205	0.96 (0.57 ,1.54)	0.845	0.96 (0.56 ,1.93)	0.887	0.63 (0.27 ,1.07)	0.159	0.41 (0.12 ,1.02)	0.097	1.3 (0.81 ,2.38)	0.301	2.31 (1.07 ,8.38)	0.107	1.14 (0.64 ,2.6)	0.714
Bifidobacterium	0.4 (0.12 ,1.21)	0.115	1 (0.38 ,2.62)	0.995	0.38 (0.11 ,1.15)	0.098	1.25 (0.45 ,3.88)	0.680	1.19 (0.46 ,3.12)	0.722	0.68 (0.25 ,1.74)	0.425	0.87 (0.26 ,3.17)	0.816	0.54 (0.19 ,1.41)	0.218	3.27 (0.95 ,13.98)	0.073	1.43 (0.53 ,3.92)	0.473	1.65 (0.63 ,4.84)	0.325	0.23 (0.05 ,0.82)	0.037
Streptococcus	3.46 (1.43 ,10.73)	0.013	0.73 (0.37 ,1.18)	0.266	1.53 (0.84 ,3.47)	0.251	0.67 (0.34 ,1.08)	0.136	0.83 (0.48 ,1.29)	0.442	0.65 (0.31 ,1.08)	0.164	1.71 (0.84 ,4.27)	0.212	0.64 (0.3 ,1.05)	0.136	1.02 (0.53 ,1.7)	0.94	1.33 (0.85 ,2.4)	0.239	2.25 (1.13 ,6.22)	0.061	1.35 (0.74 ,3.14)	0.424
Enterococcus	0.1 (0 ,0.78)	0.076	1.01 (0.57 ,1.96)	0.968	0 (0 ,0)	0.029	1.65 (0.91 ,4.19)	0.146	1.79 (0.88 ,6.61)	0.237	1.59 (0.83 ,5.01)	0.273	0 (0 ,0.03)	0.037	1.69 (0.9 ,5.05)	0.191	1.47 (0.65 ,9.34)	0.541	0.44 (0.1 ,0.94)	0.138	0.87 (0.4 ,1.55)	0.661	0 (0 ,0.1)	0.055
Deltaproteobacteria	0.07 (0 ,0.61)	0.040	1.28 (0.38 ,5.01)	0.691	0.5 (0.07 ,2.18)	0.414	3.57 (0.95 ,19.24)	0.082	1.6 (0.48 ,6.43)	0.462	1.89 (0.56 ,8.19)	0.335	0.36 (0.03 ,2.03)	0.324	1.16 (0.33 ,4.04)	0.810	0.57 (0.13 ,2.67)	0.437	0.2 (0.03 ,0.8)	0.049	0.35 (0.06 ,1.34)	0.173	0.9 (0.14 ,3.95)	0.897
Salmonella	0.82 (0.51 ,1.29)	0.383	1.21 (0.81 ,1.82)	0.347	0.82 (0.51 ,1.29)	0.383	1.47 (0.93 ,2.68)	0.133	0.8 (0.53 ,1.19)	0.281	1.08 (0.73 ,1.61)	0.699	1.06 (0.64 ,1.89)	0.831	1.17 (0.79 ,1.8)	0.441	0.39 (0.06 ,0.9)	0.13	1.35 (0.9 ,2.07)	0.153	0.89 (0.6 ,1.32)	0.562	1.05 (0.64 ,1.87)	0.847

Note: Logistic regression was utilized to assess the impact of each gut microbiota on the occurrence of various gastrointestinal and systemic symptoms.
